# Effect of Anodal Transcranial Direct Current Stimulation on Autism: A Randomized Double-Blind Crossover Trial

**DOI:** 10.1155/2014/173073

**Published:** 2014-10-30

**Authors:** Anuwat Amatachaya, Narong Auvichayapat, Niramol Patjanasoontorn, Chanyut Suphakunpinyo, Niran Ngernyam, Benchaporn Aree-uea, Keattichai Keeratitanont, Paradee Auvichayapat

**Affiliations:** ^1^Department of Physiology, Faculty of Medicine, Khon Kaen University, Khon Kaen 40002, Thailand; ^2^Department of Pediatrics, Faculty of Medicine, Khon Kaen University, Khon Kaen 40002, Thailand; ^3^Department of Psychiatry, Faculty of Medicine, Khon Kaen University, Khon Kaen 40002, Thailand

## Abstract

The aim of this study was to evaluate the Childhood Autism Rating Scale (CARS), Autism Treatment Evaluation Checklist (ATEC), and Children's Global Assessment Scale (CGAS) after anodal transcranial direct current stimulation (tDCS) in individuals with autism. Twenty patients with autism received 5 consecutive days of both sham and active tDCS stimulation (1 mA) in a randomized double-blind crossover trial over the left dorsolateral prefrontal cortex (F3) for 20 minutes in different orders. Measures of CARS, ATEC, and CGAS were administered before treatment and at 7 days posttreatment. The result showed statistical decrease in CARS score (*P* < 0.001). ATEC total was decreased from 67.25 to 58 (*P* < 0.001). CGAS was increased at 7 days posttreatment (*P* = 0.042). Our study suggests that anodal tDCS over the F3 may be a useful clinical tool in autism.

## 1. Introduction 

Autism is known as a neurodevelopmental disorder with prevalence of 62/10,000 in general population [1, 2]. The causes and pathophysiology of autism are still unclear [3]. The study by brain imaging revealed that the volume of right brain structures related to language and social function (e.g., right frontal cortex, fusiform gyrus, temporo-occipital cortex, and inferior temporal gyrus) were larger relative to their own left hemispheres or in those normal subjects [4, 5]. In addition, the abnormal function of specific brain areas (e.g., amygdala and fusiform gyrus) which participating in face processing and social cognition, have been consistently demonstrated to be hypoactivation in individual with autism spectrum disorder [6–13]. The hypoactivation of these specific brain areas, found especially at left hemisphere called rightward lateralization, were commonly evidence in individual with autism [14–17]. Several investigators have proposed that aberrant decrease in cortical plasticity may play an important role in the pathogenesis of autism [18–22]. Consistent with this hypothesis, many of the genes associated with autism are involved in various aspects of synaptic development and plasticity [23].

Up to date, there is no specific treatment for autism [24]. Behavioral therapy is suggested to be used in this therapeutic strategy [24]. However, the outcomes are still unsatisfied. In severe cases with attention deficit, pharmacologic therapies such as antidepressants and antipsychotics are recommended [25] but they may cause adverse effects such as nausea, drowsiness, dry mouth, agitation, behavioral activation, and sleep problem [25]. Therefore, there is an urgent need for more effective treatment options.

Noninvasive brain stimulation techniques, including transcranial direct current stimulation (tDCS), have been suggested as treatment options for autism [26]. tDCS involves the application of low voltage stimulation (often, 2 mA) via electrodes to the scalp. The low voltage has been shown to alter the threshold of cortical neuronal firing, such that neurons near the anode (positive lead) become more likely to fire, and neurons near the cathode (negative lead) become less likely to fire [27].

With respect to the structural- and functional-imaging paradigms, atypical rightward lateralization, and cortical plasticity mentioned above [4–22], anodal tDCS over the left hemisphere might be useful to increase the hypoactivation in individual autistic brain. This hypothesis was confirmed by the study of Schneider and colleagues; they revealed that anodal tDCS over the left dorsolateral prefrontal cortex could improve language acquisition immediately after treatment (*P* < 0.0005) and it has been hypothesized that tDCS could modulate the brain area which responds to language and cognitive function in individual with autism [28]. However, neither Childhood Autism Rating Scale (CARS) nor the Autism Treatment Evaluation Checklist (ATEC) of anodal tDCS action has been tested. Therefore, the objective of our study was to study the effects of anodal tDCS on autism parameters.

## 2. Materials and Methods

### 2.1. Participant Recruitment and Informed Consent

Study participants were recruited via advertisement at the pediatrics outpatient's neuroclinic; child development-clinic; child psychiatric clinic of Srinagarind Hospital, Faculty of Medicine, Khon Kaen University; and Khon Kaen Special Education Center Region 9, Thailand. The study procedures were described to any eligible participants who expressed an interest in participating in the study by clinic physicians. Autism diagnosis was confirmed by a child psychiatrist following a clinical review of DSM-IV TR criteria [29].

The inclusion criteria were (a) male participants with autism (b) aged between 5 and 8 with (c) mild and moderate autistic symptoms (CARS score 30–36.5). The exclusion criteria include the following: (a) on pacemaker or metallic device; (b) severe neurological disorders such as brain tumor and intracranial infection; (c) drug abuse; (d) uncooperative parents and caregivers; (e) epilepsy; (f) skull defect; and (g) use of herbal remedies and other alternative therapies.

The study was conducted in accordance with the Declaration of Helsinki and was approved by the Ethics Committee of Khon Kaen University (identifier number: HE 541409). Written informed consent was obtained from all patients and caregivers before participation.

### 2.2. Study Design

The study was a randomized double-blind controlled placebo (sham tDCS) crossover trial performed over 8 weeks consisting of (1) 1 day of baseline assessment; (2) 5 consecutive days of 1 mA anodal or sham tDCS stimulation (depending on order assignment) for 20 min; (3) 1 week of assessment; (4) 4-week washout; (5) another day of baseline assessment; (6) 5 consecutive days of 1 mA anodal or sham tDCS stimulation (depending on order assignment); and (7) a final week of outcome assessment. Thus, the study involved 8 weeks of participation. Just before the first treatment phase, participants were randomized to receive either active tDCS stimulation followed by sham stimulation or sham stimulation followed by active tDCS stimulation in a 1 : 1 ratio using a computer generated list of random numbers in blocks of four randomizations. Participants were asked to continue their routine medication regimen throughout the duration of the 8-week study.

### 2.3. Active and Sham Transcranial Direct Current Stimulation

tDCS was applied using a 35 cm^2^, 0.9% NaCl-soaked pair of surface sponge electrodes and was delivered through battery-driven power supply. The constant current stimulator had a maximum output of 10 mA (Soterix Medical, Model 1224-B, New York, USA). The anodal electrode was placed over F3 using the international 10–20 EEG electrode placement system to target the left dorsolateral prefrontal cortex (DLPFC) and the cathode electrode was placed on the right shoulder contralateral to the anode.

The tDCS device was designed to allow for masked (sham) stimulation. Specifically, the control switch was in front of the instrument, which was covered by an opaque adhesive during stimulation. The power indicator was on the front of the machine, which lit up during the time of stimulation both in active and in sham stimulations. However, in sham stimulation, the current was discontinued after 30 seconds while the power indicator remained [30].

## 3. Measures

Three main outcomes were assessed in this study: Childhood Autism Rating Scale (CARS), Autism Treatment Evaluation Checklist (ATEC), and Children's Global Assessment Scale. Moreover, the adverse events associated with active and sham stimulation procedures were also assessed.

### 3.1. Childhood Autism Rating Scale (CARS)

The CARS test is a well-established measure of autism severity [31, 32] and it was the primary outcome variable. Study participants were evaluated using a CARS test conducted by 3 investigators (NP, CS, and PA) who observed the subjects and interviewed the parent(s) and were unaware as to the treatment status of the subject. The CARS test is a 15-item behavioral rating scale developed to identify autism as well as quantitatively describe the severity of the disorder. The 15 items in the scale are the following: relating to people, imitative behavior, emotional response, body use, object use, adaptation to change, visual response, listening response, perceptive response, fear or anxiety, verbal communication, nonverbal communication, activity level, level and consistency of intellective relations, and general impressions [33]. CARS was assessed at baseline and 7-day follow-up.

### 3.2. Autism Treatment Evaluation Checklist (ATEC)

ATEC was the secondary outcome variable; the ATEC questionnaire was used to evaluate the effectiveness of treatments for autistic patients; the assessment was reported by caregiver in a total and for each of the 4 subscales as follows: (1) speech/language/communication subscale (14 items; ceiling score 28); (2) social subscale (20 items; ceiling score 40); (3) sensory and cognitive awareness subscale (18 items; ceiling score 36); and (4) health/physical/behavior problem subscale (25 items; ceiling score 75). The total score ranges from 0 to 179; a higher score indicated worsening while a lower score indicated improvement [34]. ATEC was assessed at baseline and 7-day follow-up.

### 3.3. Children's Global Assessment Scale (CGAS)

The CGAS is a global assessment of the child's psychosocial functioning [35], according to how they were described at baseline and day 7 posttreatment. The CGAS is a widely used clinician-rated scale that assigns a single summary score from 1 to 100, with 1 indicating the most severely disordered child and 100 the best-functioning child [36, 37]. Anchors at 10-point intervals include descriptors of functioning for each interval.

### 3.4. Clinical Global Impression-Improvement (CGI-I)

Overall improvement in autism was assessed using the Clinical Global Impression-Improvement (CGI-I) scale, a 7-point scale from 1 = very much improved to 7 = very much worse [38]. Scores of 1 and 2 indicate “much” or “very much” improvement and are widely considered to represent treatment success.

### 3.5. Adverse Events

Caregivers were asked to report any adverse events as well as other signs and symptoms every day after treatment. Participants were also closely observed by physicians during the stimulation session. The self-recording was terminated at one week after stimulation.

### 3.6. Statistical Analysis

For descriptive purpose, standard deviations of the demographic and outcome variables were calculated. To ensure prestimulation equivalence between participants assigned to the two treatment orders (i.e., sham-active versus active-sham), the outcome measures obtained during the first baseline period (first baseline assessment session) between these groups were compared using paired *t*-test. The dependent variables were CARS and ATEC; fixed factors were treatment order (active-sham versus sham-active), and treatment condition (active and sham condition).

Repeated measures analysis of variance (ANOVA) was used to test the hypothesis regarding the effect of tDCS on the effectiveness of treatment determined by CARS at prestimulation and 7 days after stimulation as the dependent variables, group assignment or treatment order (active-sham versus sham-active), treatment condition (active versus sham), and time (baseline and 7- day follow-up) were the independent variables.

We planned LSD to interpret any significant main or interaction effect found. A similar ANOVA procedure followed by LSD was used to test the study hypothesis regarding the effect of tDCS on CARS. CARS was assessed at prestimulation and 7 days after stimulation. All of the parameters were considered as the dependent variable, while independent variables were group assignment, treatment condition, and time. Factorial ANOVA was used to analyze the difference between the groups. The differences over time in either active or sham condition were carried out using Bonferroni correction repeated measures ANOVA.

For all analyses, *P* values less than 0.05 were considered statistically significant. Analyses were completed using Stata software, version 10.0 (StataCorp, College Station, TX).

## 4. Results

A total of 24 participants with autism were screened for possible participation between December 2012 and January 2014. Twenty individuals met the study inclusion criteria. Twelve right-handed and 8 left-handed participants completed the entire protocol. Participants assigned to each condition order did not differ significantly with respect to age, age at diagnosis, or perinatal history. Finally, no significant difference emerged between the participants assigned to each condition order in either CARS (*P* = 0.706), ATEC language subscale (*P* = 0.052), ATEC social subscale (*P* = 0.637), ATEC sensory and cognitive awareness (*P* = 0.479), ATEC health and behavioral problem (*P* = 0.387), or total ATEC score (*P* = 0.622). The age, handedness, age at diagnosis, perinatal history, and conventional treatment are presented in Table 1.

### 4.1. Childhood Autism Rating Scale Score

The CARS revealed a statistically significant amelioration of total score (*P* < 0.001; Table 2). After 7 days of anodal tDCS, the tDCS group shifted from 34.95 to 32.2. In contrast, there was no significant difference in the placebo group between baseline and 7 days posttreatment (Table 2).

### 4.2. Autism Treatment Evaluation Checklist (ATEC)

The scores of the ATEC's four subscales, as well as the total score, are presented in Table 2. There was statistical change in total ATEC score observed in the active compared to sham group (*F*(1,39) = 23.143; *P* < 0.001), as well as in health and behavioral problem (*F*(1,39) = 4.815; *P* = 0.034), sociability (*F*(1,39) = 6.525; *P* = 0.015), and sensory/cognitive awareness (*F*(1,39) = 6.171; *P* = 0.018). However, no significant change was observed in the language ATEC score (*F*(1,39) = 0.001; *P* = 0.976) at 7 days posttreatment.

### 4.3. Children's Global Assessment Scale Score

The between-group CGAS showed statistical increase in the active compared to sham group at 7 days after treatment (*P* = 0.042). Eighteen of 20 children (90%) in the active tDCS group demonstrated an increase in the score (from mean score 54.35 ± 11.07 at baseline to 60.00 ± 10.57 at the end of treatment), whereas 1 of 20 children (5%) in the sham group showed an improvement (mean score 53.35 ± 10.31 at baseline to 53.10 ± 10.14 at the end of treatment); see Table 2.

### 4.4. Clinical Global Impression-Improvement (CGI-I)

Among those who received active tDCS, only 2 children were reported to be “minimally worse,” whereas the rest were rated as “much improved” (9 of 20) and “minimally improved” (8 of 20). This gave a response rate of 85% for active tDCS. In the sham group, 3 children were rated as “much improved” to some extent, whereas 4 children were reported to have “minimally improved.” Interestingly, 7 children in the sham group were rated as “worsened” after treatment. These differences were presented in Table 2.

### 4.5. Adverse Events

Not any adverse events in participants in the active or sham groups were reported by the participants or observed by the investigators.

## 5. Discussion

To the best of our knowledge, this is the first RCT examining the effect of anodal tDCS in the treatment of patients with autism. The primary outcome revealed a significantly greater pre- to posttreatment decrease in CARS score that is maintained for 7 days among participants in the active tDCS condition relative to those in the sham tDCS condition. We also found statistically significant between-group differences in the secondary outcome variables emerged for ATEC total score at 7 days posttreatment. In addition, we found a significant CGI improvement in the active tDCS compared to in the sham group.

Since this is the first study using tDCS on CARS and ATEC, a comparison with previous results is not possible. With regard to the study using tDCS in autism, Schneider (2011) revealed a significant increase in the syntax acquisition following single dose anodal tDCS over the left dorsolateral prefrontal cortex (DLPFC) [28]. Our study did not show increase in language potential because we assessed about the comprehensive abilities while Schneider studied only in syntax.

A number of previous studies have shown some promising beneficial effects of TMS; the first study has suggested that deep rTMS to bilateral dorsomedial prefrontal cortex might yield a reduction in social relating impairment [39]. Another study of high-frequency rTMS on the left premotor cortex showed a significant increase in eye-hand performances in autistic children [40]. In addition, naming improved after rTMS of the left pars triangularis as compared with sham stimulation was observed [41].

Although the mechanisms of action of tDCS and rTMS are not fully understood, both techniques appear to produce similar changes in the activity of neuronal cell and thus may lead to similar clinical outcomes. Based on one of the autistic theories, the candidate genes in autism are involved in synaptic development and plasticity [42]. Aberrant mechanisms of plasticity can be demonstrated using TMS in patients with autism for both long term potentiation and long term depression-like plasticity [43, 44]. This postulation was confirmed by a tDCS study on increasing behavior and electrophysiology of language production [45].

Since autism is a neurodevelopmental disorder that begins in childhood and brain-derived neurotrophic factor (BDNF) is important in neurodevelopment, early BDNF hyperactivity may play a role in the pathogenesis of autism. The findings of increasing serum and brain tissue BDNF levels are presented in autism relative to normal controls. Furthermore, BDNF hyperactivity may be associated with early brain outgrowth, increase in the prevalence of seizures in autism, and similar behaviors observed in autism and fragile X syndrome [7]. In addition, it has been recently reported that tDCS, applied to mouse cortical slices, promotes long term potentiation that is absent in BDNF and TrKB mutant mice, suggesting that BDNF is a key mediator of this phenomenon [46]. This has also been demonstrated with TMS [47]. Given that assumption of an excess of BDNF related plasticity which is the rationale behind, anodal tDCS should further increase this abnormal plasticity. An important next step in research in this area is to evaluate the effect of cathodal tDCS over other associated brain areas and other potential mechanisms using BDNF as a biomarker.

An important limitation of the current study is the relatively small sample size. Thus, it may have been underpowered to detect the effects on ATEC that appeared to emerge across a number of the ATEC variables. Additional examination of tDCS's impact on ATEC in individuals with autism, ideally in studies with larger sample sizes, is warranted.

Nevertheless, despite the study's limitations, to our knowledge, this is the first study to demonstrate that anodal tDCS over the DLPFC may have beneficial effects on CARS and ATEC in individuals with autism. Further research is needed to examine these effects in larger samples of patients and to more closely examine the potential mechanisms of treatment using neuroimaging techniques.

## Figures and Tables

**Table 1:** Demographic data of participants (*n* = 20).

ID	Sex	Age (years)	Handedness	Age of diagnosis (months)	Parturition	Conventional treatment	
						Medication	Behavioral therapy
1	Male	6	Left	24	C-section	PN, RL, RD	DS, ST
2	Male	8	Right	30	C-section	—	OT
3	Male	5	Right	36	Natural	—	DS, OT
4	Male	5	Right	31	C-section	—	DS, OT
5	Male	7	Right	24	C-section	RD	DS, OT
6	Male	5	Left	32	C-section	—	DS, OT, AS (horse)
7	Male	6	Left	36	Natural	—	DS, OT, ST
8	Male	6	Left	34	Natural	—	DS, OT, ST
9	Male	8	Right	26	C-section	—	DS, OT, ST
10	Male	5	Right	24	Natural	RD	DS, ST
11	Male	7	Right	18	Natural	—	DS, ST
12	Male	6	Right	35	Natural	—	DS, OT, ST
13	Male	6	Left	35	C-section	—	DS, ST
14	Male	6	Left	29	Natural	RD	DS, ST
15	Male	8	Right	38	C-section	—	DS, ST
16	Male	7	Right	20	C-section	—	DS, OT, ST
17	Male	8	Left	40	Natural	—	DS, ST
18	Male	7	Right	36	C-section	RD	DS, OT, ST
19	Male	7	Right	32	C-section	—	DS, OT, ST
20	Male	5	Left	28	C-section	—	DS, ST

Remark: DS = developmental stimulation, ST = speech therapy, AT = animal assisted therapy, OT = occupational therapy, PN = Pyritinol, RL = Ritalin, RD = Risperidone.

**Table 2:** Childhood Autism Rating Scale (CARS), Autism Treatment Evaluation Checklist (ATEC) scale, Children's Global Assessment Scale (CGAS), Clinical Global Impression-Severity (CGI-Severity), and Clinical Global Impression-Improvement (CGI-Improvement) in the active tDCS (*n* = 20) and the sham (*n* = 20) group at baseline and 7 days posttreatment.

Parameters	Baseline				Seven days posttreatment			
	tDCS		Sham		tDCS		Sham	
	Mean	S.D.	Mean	S.D.	Mean	S.D.	Mean	S.D.
CARS	34.95	4.73	34.6	4.41	32.2^∗###^	3.98	35	4.3
ATEC								
Language	10.6	5.59	10.75	4.72	10.5	5.39	10.55	5.2
Social	16.4	4.5	17.45	2.67	14.45^∗##^	4.85	17.7	2.98
Sensory and cognitive awareness	20.1	3.91	20.5	3.4	18.35^*^	5.35	22.3	4.47
Health and behavioral problem	20.15	8.34	20.45	7.21	14.7^∗##^	6.21	19.1	6.47
Total	**67.25**	**9.88**	**69.15**	**8.98**	**58** ^∗∗∗###^	**5.82**	**69.65**	**9.13**
CGAS	54.35	11.07	53.35	10.31	60^∗###^	10.57	53.1	10.14
CGI-Severity	4.05	0.94	4.15	0.99	—	—	—	—

					*n*	%	*n*	%

CGI-Improvement								
Very much improved	—	—	—	—	0	0	0	0
Much improved	—	—	—	—	9^&^	45	3	15
Minimally improved	—	—	—	—	8	40	4	20
No change	—	—	—	—	1	5	6	30
Minimally worse	—	—	—	—	2	10	2	10
Much worse	—	—	—	—	0^&^	0	5	25
Very much worse	—	—	—	—	0	0	0	0

Mean value was significantly different between groups ^*^ *P* < 0.05, ^***^ *P* < 0.001 (one-way ANOVA), ^&^ *P* < 0.05 (chi-square test).

Mean value was significantly different from that at baseline ^##^ *P* < 0.01, ^###^ *P* < 0.001 (paired *t*-test).
